# Supplementary material for: Splice-Junction-Based Mapping of Alternative Isoforms in the Human Proteome
Source: Cell Rep. Author manuscript; Available in PMC 2020 Jan 15. (PMC6961840; doi:10.1016/j.celrep.2019.11.026)
Supplement: 2 [file NIHMS1546469-supplement-2.zip › DF1/PXD009737/TestisTrypsin/TestisTrypsin_78_RRNAD1_QPMGHTPLLMAGSIGWTE.pdf]

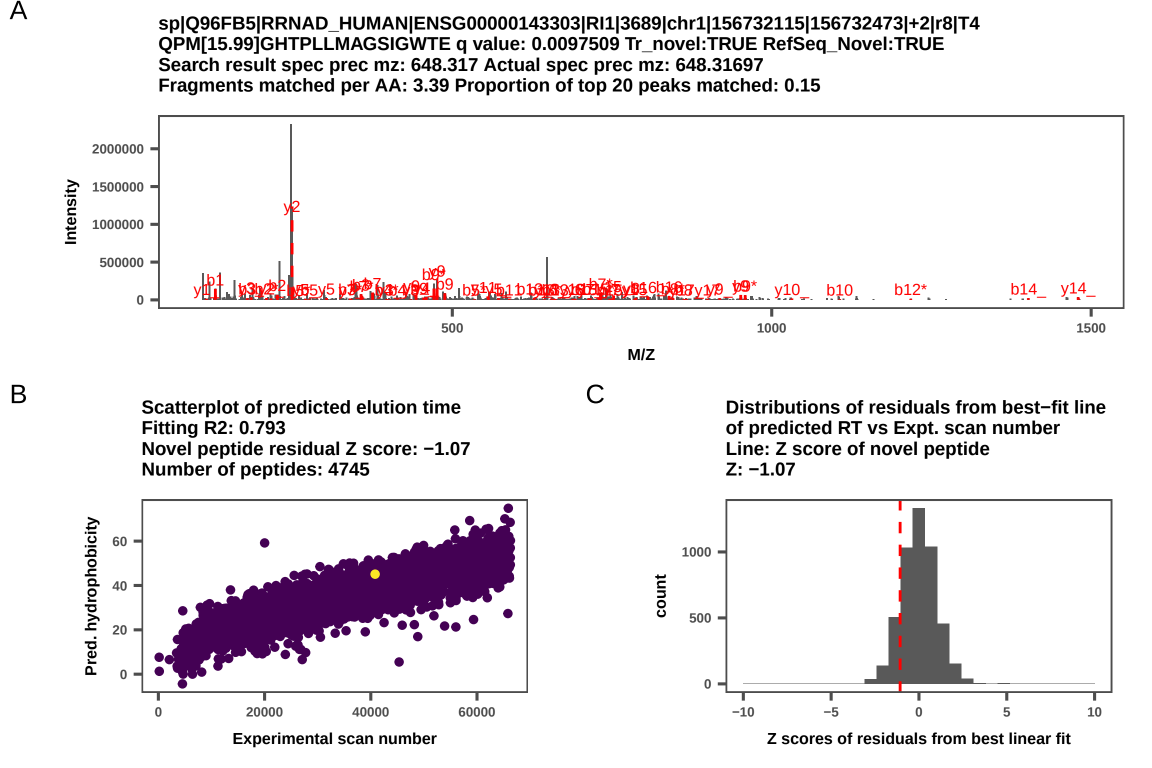
A
sp|Q96FB5|RRNAD_HUMAN|ENSG00000143303|RI1|3689|chr1|156732115|156732473|+2|r8|T4
QPM[15.99]GHTPLLMAGSIGWTE q value: 0.0097509 Tr_novel:TRUE RefSeq_Novel:TRUE
Search result spec prec mz: 648.317 Actual spec prec mz: 648.31697
Fragments matched per AA: 3.39 Proportion of top 20 peaks matched: 0.15
Intensity
2000000
1500000
1000000
500000
0
y2
y1
b1
b2
b9
y9
b9
y10_
b10
b12*
b14_
y14_
500
1000
1500
M/Z
B
Scatterplot of predicted elution time
Fitting R2: 0.793
Novel peptide residual Z score: −1.07
Number of peptides: 4745
Pred. hydrophobicity
60
40
20
0
0
20000
40000
60000
Experimental scan number
C
Distributions of residuals from best−fit line
of predicted RT vs Expt. scan number
Line: Z score of novel peptide
Z: −1.07
count
1000
500
0
−10
−5
0
5
10
Z scores of residuals from best linear fit
